# No association between dietary magnesium intake and body composition among Iranian adults: a cross-sectional study

**DOI:** 10.1186/s40795-022-00535-6

**Published:** 2022-04-29

**Authors:** Amin Mirrafiei, Bahareh Jabbarzadeh, Yasaman Hosseini, Kurosh Djafarian, Sakineh Shab-Bidar

**Affiliations:** 1 Department of Community Nutrition, School of Nutritional Sciences and Dietetics, Tehran University of Medical Sciences (TUMS), Tehran, 14167-53955 Iran; 2 Department of Clinical Nutrition, School of Nutritional Sciences and Dietetics, Tehran University of Medical Sciences (TUMS), Tehran, 14167-53955 Iran

**Keywords:** Magnesium, Diet, Body Composition, Obesity

## Abstract

**Purpose:**

Obesity is becoming more prevalent worldwide. Magnesium (Mg) intake may play a role in the regulation of energy metabolism and body weight. Therefore, in this cross-sectional study, we aimed to investigate the association between dietary Mg intake and body composition among healthy adults.

**Methods:**

A total of 778 adult men and women aged 18–59 years who attended health care centers in Tehran, Iran, entered the final analysis. Dietary intake was assessed with a validated and reliable food frequency questionnaire with 168 items and the dietary Mg intake was estimated using Nutritionist IV software. Anthropometric measurements and blood samples were collected and body composition was evaluated employing the Body Mass Index (BMI), A Body Shape Index (ABSI), Body Adiposity Index (BAI), Body Roundness Index (BRI), Visceral Adiposity Index (VAI), Lipid Accumulation Index (LAP), and Triglyceride-Glucose index (TyG). Multiple linear regression analysis was used to determine the association of the dietary Mg intake with body composition indices.

**Results:**

The mean daily dietary Mg intake was 294 ± 140 mg in men and 262 ± 112 mg in women. Unadjusted linear regression showed that dietary magnesium intake is significantly associated with a waist to hip ratio (WHR) and total cholesterol (TC) in men, and hip circumference (HC) in women. After adjusting for potential confounders including age, education, marriage, occupation and smoking, total energy intake, and activity score, there remained no significant association between dietary Mg intake and any of the body composition indices including BMI, ABSI, BAI, BRI, VAI, LAP, and TyG neither in men nor women.

**Conclusion:**

Higher Mg intake was not associated with anthropometric indices in Iranian adults, according to our findings. Additional observational studies would be beneficial in clarifying the existing findings.

## Background

In the past two decades, the prevalence of obesity has increased almost 12% and has reached a global record of 42.4% [1]. It may lead to heart diseases [2], different types of cancer [3], type 2 diabetes [4], and strokes [5], which are the primary causes of worldwide premature death [6]. The problem is more severe in developing countries that have a weaker health control system. In Iran, the prevalence of overweight and obesity were 41% and 13% in 2019, respectively, and is following an increasing trend [7].

Body Mass Index (BMI) is the most common way to evaluate body composition and obesity [8], but it does not differentiate between fat mass and fat-free mass or the location of the body fat and is not inferable to different ethnicities [9]. Therefore, other indices are necessary to assess body composition precisely. Fat distribution is as important as the fat mass in predicting risk factors [10]. Visceral Adiposity Index (VAI), A body Shape Index (ABSI), Body Roundness Index (BRI), Lipid Accumulation Index (LAP), and Body Adiposity Index (BAI) are all fairly novel indices that may enhance the measurement of obesity and body composition These indices have all been currently considered reliable and valid, and have strengths and limitations compared to each other (15-11).

Besides excessive energy intake, obesity and the poor shape of the body might be the result of inadequate nutrient consumption, as most obese people have a low intake of vital minerals and vitamins [11, 12]. The association of several micronutrients including calcium, zinc, iodine, selenium, with body composition has been studied [13–16]. Of these, magnesium (Mg) may play a key role in energy metabolism and obesity. Mg is involved in more than 300 biochemical reactions in the human body. Synthesis of proteins, muscle and nerve transmission, blood glucose control, and regulation of blood pressure have all been linked to Mg [17, 18]. It has been observed in some studies that there is a negative association between Mg intake and markers of obesity, such as waist circumference (WC) and BMI [19–21]. It may theoretically be the result of the regulatory effect of the referred mineral on blood glucose level and insulin resistance [22] or the synthesis of fatty acids [23]. Mg is essential for activating several key enzymes of glucose pathways and therefore, Mg deficiency can change the oxidative metabolism of glucose [24]. A decrease of the activity of Mg in the liver might generate an excess amount of NADPH from glucose thus, causing an expansion in the synthesis of TG and LDL, which the former would go on to store in adipocytes and promote adiposity [25]. Apart from this, Mg act as the cofactor of enzyme cholesterol acyltransferase (CAT) and lipoprotein lipase (LPL), two enzymes participating in fat metabolism. On the other hand, a non-significant relation has been discovered in other studies [26, 27].

Although the results can be generalizable to different geographic populations, there is still limited evidence of the mentioned association in populated developing countries like Iran, where the consumption of whole grains, legumes, and nuts, the main sources of Mg are low [28]. As obesity is a quite clear phenomenon in middle Eastern people including Iran, the aim of the current cross-sectional study is to investigate the association of magnesium intake, exclusively from the diet, and body composition and anthropometric indices in a sample of Iranian adults.

## Materials and methods

### Study design

A total of 850 healthy adult men and women, aged 18 to 59, who were willing to participate in this cross-sectional study, were recruited from health care centers of Tehran, from 2018 to 2019, via a two-stage cluster sampling using advertisement, distribution of flyers in common places and information sessions at health care centers about the goal and the benefit of the examination. First, the city was split into five regions north, east, south, west, and center. A list of all existing health care centers was provided and then eight health centers were randomly chosen from each region for a tally of forty health centers. Ultimately, the sample size (*n* = 850) was divided by 40 to obtain the number of subjects in each health center. Based on the prevalence of obesity and overweight in the adults of Tehran (65%), an error coefficient of d = 0.04 and at α level of 0.05, a sample size of 546 people was calculated.$\left(\text{n}\mathit=\frac{z^2\mathit-p\text{(1}\mathit-p\mathit)}{d^2}\frac{{(1.96)}^2\ast0.65\ast0.35}{{(0.04)}^2}\right)\;$  Due to the potential exclusion of participants, the sample size was multiplied by 1.5 which included the total number of 850 subjects. Of these, 72 participants were excluded, 50 of them because of missing data, 13 because of under-reporting, and 9 for the reason of over-reporting of energy intake (800 > , 4000 <) [29]. We conducted the final analysis on 778 subjects.

### Data collection

Information about sex (male/ female), age (year), educational level (under diploma/ diploma and higher), smoking status (never or former smoker/ current smoker), occupation (employed/ house keeper/ retired/ unemployed) and marital status (single/ married/ divorced) were collected via demographic questionnaire during the initial visit.

Physical activity was assessed using the short form of the International physical activity questionnaire (IPAQ) [30], consisting of 7 validated questions. Data was collected regarding walking, moderate, and vigorous activity, in the previous week, and a physical activity level was determined within two categories of metabolic equivalents (METs) [31], as low (< 600 MET-minutes/week) or moderate and high physical activity (> 600 MET-minutes per week).

Systolic (SBP) and diastolic blood pressure (DBP) were measured twice by a standard mercury sphygmomanometer (BC 08; Beurer, Ulm, Germany), on the right arm, after 15 min of resting, performed by a trained physician. The second measurement was done 1–2 min later. The mean of the two measurements was reported as the blood pressure of each individual.

### Magnesium intake assessment

The dietary intake of the participants was assessed using a validated and reliable semi-quantitative food frequency questionnaire (FFQ) with 168 food items [32], which consists of a list of foods with a standard serving size commonly consumed and estimates the mean intake of each food on a daily, weekly, monthly, or annual basis. It was administered by an experienced dietician through face-to-face interviews. Energy and nutrient intake estimated with FFQ has been proven to be valid and accurate [32]. Portion sizes of the consumed foods were converted to grams per day [33] and intake of energy and Mg content of foods were estimated using the Nutritionist IV software based on the Iranian foods-modified US Department of Agriculture food composition [34].

### Anthropometric measurements

The height of the participants was calibrated using a wall stadiometer with a precision of 0.1 cm while standing in a normal position with no shoes on (Seca, Germany). Weight was measured by a digital scale with a sensitivity of 100 g (Seca808; Seca, Hamburg, Germany), with minimum clothes on. WC was assessed utilizing a tape measure between the lowest rib and Iliac crest during exhalation. HC was measured by a non-stretch tape around the widest portion of the buttocks over light clothing without any pressure on the body surface and WHR was calculated. To minimize subjective errors, a single technician did all the measurements.

Following mathematical equations were used to measure anthropometric indices:

$$BMI\hspace{0.17em}=\hspace{0.17em}\frac{Weight (kg)}{{Height (m)}^{2}}$$

The most widely used index for anthropometric measurement in epidemiological research traditionally classifies obesity. It is regarded as a reliable guideline for weight management for many years [35].

$$BAI\hspace{0.17em}=\hspace{0.17em}\frac{100 \times HC \left(m\right)}{Height(m) \times \sqrt{Height }(m)} -18$$

This parameter was defined by Bergman et al. and can evaluate adiposity percent directly even in the clinical and critical situations using only HC and height. Dissimilar to BMI, the BAI can predict the percentage of body fat in both men and women without statistical correction [36].

$$BRI\hspace{0.17em}=\hspace{0.17em}364.2\hspace{0.17em}-\times \hspace{0.17em}\surd (1-(\frac{{\left(WC (cm)/2\pi \right)}^{2}}{{\left(0.5 \times Height (cm)\right)}^{2}}))$$

Instituted by Thomas et al., it is a predictor of body fat and visceral adiposity fat percent that can assess health status [37]. It has been investigated that BRI is one of the most accurate indices in predicting metabolic syndrome among adults [38].

$$ABSI\hspace{0.17em}=\hspace{0.17em}\frac{WC (m)}{{BMI (\frac{kg}{{m}^{2}})}^\frac{2}{3}\times {Height (m)}^\frac{1}{2}}$$

First introduced by Krakauer et al. and based on the same principle used for designing BMI, ABSI measures body shape, as a risk factor for premature death and all-cause mortality in the general population and is considered one of the best indices for measuring abdominal obesity [39, 40].

$$LAP (men)\hspace{0.17em}=\hspace{0.17em}\left(Waist \left(cm\right)-65\right)\times TG (\frac{mmol}{l})$$

$$LAP (women)\hspace{0.17em}=\hspace{0.17em}\left(Waist \left(cm\right)-58\right)\times TG (\frac{mmol}{l})$$

LAP is considered to be a mighty index that predicts insulin resistance (IR) in the non-diabetic population better than BMI. It is an easy index associated with lipid overaccumulation which can detect IR in large populations [41, 42]. It is a more reliable predictor of cardiovascular risk [43].

$$VAI (men)\hspace{0.17em}=\hspace{0.17em}(\frac{WC (cm)}{39.68+\left(1.88\times BMI\right)})\times (\frac{TG (\frac{mmol}{l})}{1.03})\times (\frac{1.31}{HDL (\frac{mmol}{l})})$$

$$VAI (women)\hspace{0.17em}=\hspace{0.17em}(\frac{WC (cm)}{36.58+\left(1.89\times BMI\right)})\times (\frac{TG (\frac{mmol}{l})}{0.81})\times (\frac{1.52}{HDL (\frac{mmol}{l})})$$

A sex-specific index, based on WC, BMI, Triglyceride (TG), and High-Density Lipoprotein (HDL), that indirectly calculates visceral fat function and distribution. VAI was introduced by Amato et al. and was able to estimate the association between visceral adiposity dysfunction and cardiometabolic risk [44].

$$TyG\hspace{0.17em}=\hspace{0.17em}Ln[TG(\frac{mg}{dl})\times \frac{FPG\left(\frac{mg}{dl}\right)}{2}]$$

Triglyceride-Glucose index (TyG) is a novel marker, which has been revealed to have a high sensitivity and specificity in recognizing metabolic syndrome, cardiovascular risk, and insulin resistance at the early stages [45].

### Biochemical measurements

Blood samples were collected after 12 h of fasting since the previous night by approved methods at the Nutrition and Biochemistry Laboratory of the School of Nutritional Sciences and Dietetics at Tehran University of Medical Sciences and the serum levels of TG, Fasting Plasma Glucose (FPG), and HDL were determined.

### Statistical analysis

General characteristics of the participants were described using mean and standard deviation (SD). Since total energy that one person consumes plays a defining role on micronutrient intake, dietary Mg intake (mg) was adjusted for the total energy intake (kcal), using the residual method performed by the linear regression, with Mg intake, exclusively from the diet, as the dependent variable and total energy intake as the independent variable, and then got categorized into quartiles. To compare the means of different quantitative and qualitative variables across quartiles of adjusted Mg intake and in each sex, we applied a one-way analysis of variance (ANOVA) and chi-squared test. Analysis of covariance (ANCOVA) was applied to assess the means of biochemical and anthropometric parameters across quartiles of the energy-adjusted Mg intake for each sex adjusting for age, marital status, occupation, education status, smoking status, physical activity, and energy intake. Multiple linear regression with a confidence interval of 95 percent was used to determine the relationship between dietary Mg intake and anthropometric indices and blood glucose and lipids values, controlling for age, age, educational level, marital status, occupation, smoking, activity score, and total energy intake as covariates, in each gender separately. All analyses were run by using SPSS software (SPSS Inc., version 26), and p < 0.05 was defined as significant.

## Results

The current cross-sectional study included 778 participants, 546 women and 266 men aged 18 to 59 years, with a mean age of 45.7 years in men and 44.6 years in women. Men significantly had higher energy intake (mean ± SD: 2441 ± 682) and dietary Mg consumption (mean ± SD: 294 ± 140) than women (mean ± SD: 2228 ± 751 and 262 ± 112), respectively (*P* =  < 0.001). Other characteristics of the study population, classified by sex, and the quartiles of energy-adjusted Mg intake, are presented in Table 1 and Table 2.

**Table 1:** General characteristics of study participants by sex

	**Total (*n* = 778)**	**Men (*n* = 232)**	**Women (*n* = 546)**	*p*- value*
**Age**	44.9 ± 10.6	45.7 ± 9.73	44.6 ± 11.0	0.22
**Education**				0.02
Under diploma	284 (36.5%)	88 (38%)	194 (35.5%)	
Diploma and higher	494 (63.5%)	144 (62%)	352 (64.5%)	
**Smoking status**				< 0.001
Never or former smoker	739 (95%)	204 (88%)	535 (98%)	
Current smoker	39 (5%)	28 (12%)	11 (2%)	
**Physical activity**				0.32
Low	490 (63%)	138 (59.5%)	352 (64.5%)	
Moderate and higher	288 (37%)	94 (40.5%)	194 (35.5%)	
**Occupation**				< 0.001
Employee	189 (24.3%)	91 (39.2%)	98 (18%)	
Housekeeper	449 (57.7%)	70 (30.2%)	379 (69.4%)	
Retired	116 (14.9%)	69 (29.7%)	47 (8.6%)	
Unemployed	24 (3.1%)	2 (0.9%)	22 (4%)	
**Marital status**				< 0.001
Single	80 (10.3%)	19 (8.2%)	61 (11.3%)	
Married	635 (81.6%)	210 (90.5%)	425 (77.9%)	
Divorced	63 (8.1%)	3 (1.3%)	60 (11%)	
**Energy**	2291 ± 737	2441 ± 682	2228 ± 751	< 0.001
** Total Mg intake (mg/day)**	272 ± 122	294 ± 140	262 ± 112	< 0.001
** Magnesium intake/ 1000 kcal**	125 ± 58.9	131 ± 76.2	123 ± 49.6	0.10

Values are mean ± standard deviation for continuous variables and quantity and percent for categorical variables

The *p* values resulted from the analysis of one-way analysis of variance for continuous variables and χ2 test for categorical variables

^*^The *p* < 0.05 is significant

**Table 2:** General characteristics of study participants by energy-adjusted quartiles of Mg intake

	**1**^**st**^** quartile (193)**	**2**^**nd**^** quartile (196)**	**3**^**rd**^** quartile (195)**	**4**^**th**^** quartile (194)**	*p*- value*
**Age**	45.7 ± 10.2	44.2 ± 10.7	43.4 ± 10.9	46.6 ± 10.4	0.01
**Female %**	60.6	75.0	76.9	68.0	0.01
**Education**					0.16
Under diploma	74 (38.3%)	68 (34.7%)	64 (32.9%)	76 (39.2%)	
Diploma and higher	119 (61.7%)	128 (65.3%)	131 (67.1%)	118 (60.8%)	
**Smoking status**					0.04
Never or former smoker	179 (92.7%)	191 (97.4%)	188 (96.4%)	181 (93.3%)	
Current smoker	14 (7.3%)	5 (2.6%)	7 (3.6%)	13 (6.7%)	
**Physical activity**					0.69
Low	121 (62.7%)	123 (62.8%)	119 (61.0%)	127 (65.5%)	
Moderate and higher	72 (37.3%)	73 (37.2%)	76 (39.0%)	67 (34.5%)	
**Occupation**					0.01
Employee	49 (25.4%)	54 (27.6%)	52 (26.7%)	34 (17.5%)	
Housekeeper	111 (57.5%)	112 (57.1%)	110 (56.4%)	116 (59.8%)	
Retired	33 (17.1%)	20 (10.2%)	23 (11.8%)	40 (20.6%)	
Unemployed	0 (0%)	10 (5.1%)	10 (5.1%)	4 (2.1%)	
**Marital status**					0.16
Single	14 (7.3%)	25 (12.8%)	24 (12.3%)	17 (8.8%)	
Married	167 (86.5%)	154 (78.5%)	159 (81.5%)	155 (79.9%)	
Divorced	12 (6.2%)	17 (8.7%)	12 (6.2%)	22 (11.3%)	
**Energy**	2818 ± 691	2247 ± 585	2121 ± 704	1985 ± 681	< 0.001
** Total Mg intake (mg/day)**	200 ± 48.3	232 ± 61.9	269 ± 90.3	385 ± 160	< 0.001
** Magnesium intake/ 1000 kcal**	72.6 ± 13.1	103 ± 6.36	127 ± 7.72	198 ± 71.3	< 0.001

Values are mean ± standard deviation for continuous variables and quantity and percent for categorical variables

The *p* values resulted from the analysis of one-way analysis of variance for continuous variables and χ2 test for categorical variables

^*^The *p* < 0.05 is significant

Table 3 shows the mean energy and dietary intake of Mg, anthropometric indices, and blood glucose and lipids across the quartiles of energy-adjusted dietary Mg consumption in men, adjusted for age, marital status, occupation, education status, smoking status, physical activity, and energy intake. Men in the highest quartile of energy-adjusted Mg intake had a significantly lower total daily energy intake compared to the lowest quartile (*P* < 0.001). None of the other values were significantly different across the quartiles. Alternatively, Table 4 presents the same values among women of the study. Similarly, women in the top quartile of energy-adjusted Mg intake had a significantly lower total daily energy intake compared to the first quartile (*P* < 0.001). Also, there was a significant decrease in BRI across the quartiles (*P* = 0.03).

**Table 3:** Multivariate-adjusted Sociodemographic, body composition indices and biochemical factors across quartiles of dietary Mg intake in Iranian men

	**1**^**st**^** quartile**	**2**^**nd**^** quartile**	**3**^**rd**^** quartile**	**4**^**th**^** quartile**	*p*-value*
Mg intake (mg/day)	212 ± 11.6	241 ± 14.6	284 ± 15.1	447 ± 13.0	< 0.001
Total energy intake (Kcal)	2942 ± 65.9	2351 ± 82.9	2248 ± 86.0	2039 ± 74.2	< 0.001
Weight (Kg)	79.7 ± 1.68	79.8 ± 1.91	80.8 ± 1.97	81.1 ± 1.79	0.94
BMI (kg/m^2^)	27.8 ± 0.49	27.1 ± 0.56	27.9 ± 0.58	27.6 ± 0.53	0.73
WHR	0.92 ± 0.01	0.92 ± 0.01	0.92 ± 0.01	0.95 ± 0.01	0.19
WC (cm)	94.8 ± 1.39	92.4 ± 1.59	93.8 ± 1.64	97.6 ± 1.49	0.10
HC (cm)	102 ± 1.11	99.8 ± 1.26	102 ± 1.30	103 ± 1.19	0.23
SBP (mm Hg)	119 ± 2.70	120 ± 3.07	126 ± 3.18	122 ± 2.89	0.35
DBP (mm Hg)	77.3 ± 1.79	78.8 ± 2.04	80.0 ± 2.11	80.8 ± 1.92	0.63
BAI	28.6 ± 0.55	26.7 ± 0.61	28.1 ± 0.64	28.2 ± 0.58	0.11
BRI	4.69 ± 0.16	4.26 ± 0.18	4.50 ± 0.19	4.85 ± 0.18	0.11
ABSI	0.08 ± 0.00	0.08 ± 0.00	0.08 ± 0.00	0.08 ± 0.00	0.23
VAI	1.79 ± 0.15	1.54 ± 0.17	2.02 ± 0.18	1.96 ± 0.16	0.22
TyG Index	8.80 ± 0.07	8.74 ± 0.08	8.89 ± 0.08	8.85 ± 0.08	0.64
LAP index	48.1 ± 4.06	39.3 ± 4.56	50.7 ± 4.78	54.8 ± 4.34	0.10
HDL (mg/dl)	49.1 ± 1.28	51.0 ± 1.43	49.2 ± 1.50	48.5 ± 1.36	0.63
TC (mg/dl)	187 ± 5.86	201 ± 6.58	200 ± 6.88	187 ± 6.25	0.27
TG (mg/dl)	146 ± 9.34	129 ± 10.5	158 ± 11.0	147 ± 9.97	0.22
FBG (mg/dl)	106 ± 3.65	109 ± 4.10	106 ± 4.29	107 ± 3.90	0.96

^*^P-value is considered significant at < 0.05, Obtained from ANCOVA, adjusted for age, occupation, education, smoking, physical activity, marriage and energy intake (except itself)

Values are mean ± standard error

Abbreviations: *Mg* Magnesium, *BMI* Body Mass Index, *WHR* Waist to Hip ratio, *WC* Waist circumference, *HC* Hip Circumference, *SBP* Systolic Blood Pressure, *DBP* Diastolic Blood Pressure, *BAI* Body Adiposity Index, *BRI* Body Roundness Index, *ABSI* A Body Shape Index, *HDL* High-Density Lipoprotein, *TC* Total Cholesterol, *TG* Triglyceride, *VAI* Visceral Adiposity Index, *FBG* Fast Blood Sugar, *TyG* Triglyceride Glucose Index, *LAP* Lipid Accumulation Product

**Table 4:** Multivariate-adjusted Sociodemographic, body composition indices and biochemical factors across quartiles of dietary Mg intake in Iranian women

	**1**^**st**^** quartile**	**2**^**nd**^** quartile**	**3**^**rd**^** quartile**	**4**^**th**^** quartile**	*p*-value*
Mg intake (mg/day)	195 ± 8.94	229 ± 7.94	264 ± 7.88	356 ± 8.40	< 0.001
Total energy intake (Kcal)	2749 ± 64.6	2203 ± 57.4	2069 ± 57.0	1975 ± 60.7	< 0.001
Weight (Kg)	69.6 ± 1.15	70.6 ± 0.96	70.5 ± 0.96	69.7 ± 1.03	0.87
BMI (kg/m^2^)	27.8 ± 0.44	27.9 ± 0.37	27.7 ± 0.37	27.8 ± 0.40	0.96
WHR	0.88 ± 0.01	0.86 ± 0.01	0.86 ± 0.01	0.86 ± 0.01	0.03
WC (cm)	92.4 ± 1.04	89.3 ± 0.87	90.0 ± 0.87	91.4 ± 0.93	0.10
HC (cm)	104 ± 1.05	104 ± 0.88	105 ± 0.88	106 ± 0.95	0.57
SBP (mm Hg)	120 ± 1.95	118 ± 1.64	118 ± 1.63	122 ± 1.76	0.28
DBP (mm Hg)	77.5 ± 1.31	78.2 ± 1.10	76.6 ± 1.10	79.8 ± 1.18	0.25
BAI	34.5 ± 0.59	34.2 ± 0.49	34.0 ± 0.49	35.3 ± 0.53	0.31
BRI	5.27 ± 0.16	4.74 ± 0.13	4.78 ± 0.13	5.08 ± 0.14	0.03
ABSI	0.08 ± 0.00	0.08 ± 0.00	0.08 ± 0.00	0.08 ± 0.00	0.06
VAI	2.47 ± 0.18	2.88 ± 0.15	2.59 ± 0.15	2.46 ± 0.16	0.19
TyG Index	8.79 ± 0.06	8.86 ± 0.05	8.77 ± 0.05	8.72 ± 0.05	0.21
LAP index	53.0 ± 3.29	55.5 ± 2.76	51.6 ± 2.76	51.4 ± 2.96	0.71
HDL (mg/dl)	50.2 ± 0.99	49.9 ± 0.83	49.0 ± 0.83	51.1 ± 0.89	0.38
TC (mg/dl)	196 ± 4.30	201 ± 3.61	195 ± 3.60	197 ± 3.87	0.70
TG (mg/dl)	137 ± 7.25	156 ± 6.09	141 ± 6.08	136 ± 6.54	0.09
FBG (mg/dl)	109 ± 2.61	108 ± 2.19	107 ± 2.18	101 ± 2.35	0.08

^*^P-value is considered significant at < 0.05, Obtained from ANCOVA adjusted for occupation, education, smoking, physical activity, marriage and energy intake (except itself)

Values are mean ± standard error

Abbreviations: *Mg* Magnesium, *BMI* Body Mass Index, *WHR* Waist to Hip ratio, *WC* Waist circumference, *HC* Hip Circumference, *SBP* Systolic Blood Pressure, *DBP* Diastolic Blood Pressure, *BAI* Body Adiposity Index, *BRI* Body Roundness Index, *ABSI* A Body Shape Index, *HDL* High-Density Lipoprotein, *TC* Total Cholesterol, *TG* Triglyceride, *VAI* Visceral Adiposity Index, *FBG* Fast Blood Sugar, *TyG* Triglyceride Glucose Index, *LAP* Lipid Accumulation Product

Crude and multivariable-adjusted beta coefficients (β) with 95 percent confidence intervals of anthropometric indices and blood glucose and lipids across quartiles of the dietary Mg intake, as a continuous variable, among men and women are presented in Table 5 and Table 6. No significant association was observed between dietary Mg intake and body composition factors in men and women neither in any of the models, although there were some exceptions. There was a significant association for WHR in men, which a higher dietary Mg intake was associated with a higher WHR (β:0.001, 95% CI:0.000–0.001, *P* = 0.03) and TC (β: -0.044, 95% CI: -0.086- -0.002, *P* = 0.04) in the non-adjusted model. In women, higher intake was significantly associated with a higher BAI in partially-adjusted (β:0.005, 95% CI:0.000–0.009, *P* = 0.04), there was a higher likelihood of increased WC in the second model (β:0.009, 95% CI:0.001–0.017, *P* = 0.03), and finally, we observed an elevated HC with higher dietary Mg intake in both non-adjusted (β:0.012, 95% CI:0.004–0.020, *P* = 0.01) and partially-adjusted model (model 2; β:0.012, 95% CI:0.004–0,020, *P* = 0.01).

**Table 5:** Association of dietary magnesium intake with anthropometric indices and biochemical factors in Iranian men

Magnesium intake (mg)				
	**β ± SE**	**R**^**2**^	**95% CI**	*P* value*
**LAP**				
Model 1	0.015 ± 0.015	0.000	-0.015,0.044	0.33
Model 2	0.013 ± 0.015	0.000	-0.017,0.043	0.39
Model 3	0.013 ± 0.015	-0.001	-0.018,0.043	0.41
**BRI**				
Model1	0.001 ± 0.001	0.006	-0.000,0.002	0.13
Model 2	0.000 ± 0.001	0.162	-0.001,0.001	0.74
Model 3	0.000 ± 0.001	0.162	-0.001,0.001	0.77
**ABSI**				
Model 1	0.000 ± 0.000	0.008	-0.000,0.000	0.10
Model 2	0.000 ± 0.000	0.070	-0.000,0.000	0.39
Model 3	0.000 ± 0.000	0.066	-0.000,0.000	0.41
**BAI**				
Model 1	0.001 ± 0.002	-0.004	-0.004,0.005	0.74
Model 2	-0.001 ± 0.002	0.129	-0.005,0.003	0.59
Model 3	-0.001 ± 0.002	0.122	-0.005,0.003	0.58
**VAI**				
Model 1	0.000 ± 0.001	-0.003	-0.001,0.001	0.58
Model 2	0.000 ± 0.001	0.002	-0.001,0.002	0.40
Model 3	0.000 ± 0.001	-0.001	-0.001,0.002	0.40
**TyG**				
Model 1	0.000 ± 0.000	-0.004	-0.000,0.001	0.90
Model 2	0.000 ± 0.000	0.010	-0.000,0.001	0.60
Model 3	0.000 ± 0.000	0.004	-0.000,0.001	0.61
**HDL (mg/dl)**				
Model 1	-0.005 ± 0.003	0.001	-0.009,0.003	0.38
Model 2	-0.006 ± 0.003	0.009	-0.009,0.003	0.36
Model 3	-0.006 ± 0.003	0.006	-0.009,0.003	0.37
**TC (mg/dl)**				
Model 1	-0.044 ± 0.005	0.021	-0.086, -0.002	0.04
Model 2	-0.036 ± 0.005	0.022	-0.079, 0.007	0.10
Model 3	-0.036 ± 0.005	0.022	-0.079, 0.008	0.11
**TG (mg/dl)**				
Model 1	-0.005 ± 0.034	-0.004	-0.073,0.062	0.88
Model 2	0.009 ± 0.035	0.000	-0.060,0.078	0.79
Model 3	0.009 ± 0.035	-0.001	-0.060,0.078	0.80
**FBS (mg/dl)**				
Model 1	0.006 ± 0.013	-0.003	-0.020,0.033	0.63
Model 2	0.009 ± 0.014	-0.004	-0.018,0.036	0.53
Model 3	0.008 ± 0.014	0.024	-0.019,0.035	0.54
**WC (cm)**				
Model 1	0.010 ± 0.005	0.010	-0.001,0.020	0.07
Model 2	0.005 ± 0.005	0.105	-0.005,0.016	0.31
Model 3	0.005 ± 0.005	0.112	-0.005,0.015	0.33
**HC (cm)**				
Model 1	0.003 ± 0.004	-0.002	-0.005,0.011	0.46
Model 2	0.002 ± 0.004	0.082	-0.006,0.010	0.60
Model 3	0.002 ± 0.004	0.078	-0.006,0.010	0.62
**WHR**				
Model 1	0.001 ± 0.000	0.017	0.000,0.001	**0.03**
Model 2	0.000 ± 0.000	0.110	-0.000,0.000	0.21
Model 3	0.000 ± 0.000	0.121	-0.000,0.000	0.22
**BMI (kg/m**^**2**^**)**				
Model 1	0.000 ± 0.002	-0.004	-0.003,0.004	0.84
Model 2	-0.000 ± 0.002	0.016	-0.004,0.004	0.99
Model 3	-0.000 ± 0.002	0.014	-0.004,0.004	0.97

Values are β coefficients (95% CIs), *n* = 232. Results are from multiple linear regression analyses

Model 1: crude

Model 2: adjusted for age, education, marriage, occupation and smoking

Model 3: additionally, adjusted for energy intake and activity score

^*^P-value is considered significant at < 0.05

Abbreviations: *SD* Standard Deviation, *BMI* Body Mass Index, *WHR* Waist to Hip ratio, *WC* Waist circumference, *HC* Hip Circumference, *SBP* Systolic Blood Pressure, *DBP* Diastolic Blood Pressure, *BAI* Body Adiposity Index, *BRI* Body Roundness Index, *ABSI* A Body Shape Index, *HDL* High-Density Lipoprotein, *TC* Total Cholesterol, *TG* Triglyceride, *VAI* Visceral Adiposity Index, *FBG* Fast Blood Sugar, *TyG* Triglyceride Glucose Index, *LAP* Lipid Accumulation Product

**Table 6:** association of dietary magnesium intake with anthropometric indices and biochemical factors in Iranian women

Magnesium intake (mg)				
	**β ± SE**	**R**^**2**^	**95% CI**	P value*
**LAP**				
Model 1	0.009 ± 0.013	-0.001	-0.017,0.036	0.49
Model 2	0.011 ± 0.013	0.076	-0.015,0.036	0.41
Model 3	-0.006 ± 0.015	0.089	-0.034,0.023	0.70
**BRI**				
Model1	0.001 ± 0.001	0.000	-0.001,0.002	0.26
Model 2	0.001 ± 0.001	0.294	-0.000,0.002	0.15
Model 3	-0.000 ± 0.001	0.305	-0.002,0.001	0.80
**ABSI**				
Model 1	0.000 ± 0.000	-0.002	-0.000,0.000	0.75
Model 2	0.000 ± 0.000	0.053	-0.000,0.000	0.71
Model 3	-0.000 ± 0.000	0.060	-0.000,0.000	0.38
**BAI**				
Model 1	0.005 ± 0.003	0.004	-0.000,0.010	0.06
Model 2	0.005 ± 0.002	0.199	0.000,0.009	**0.04**
Model 3	-0.002 ± 0.003	0.202	-0.003,0.008	0.35
**VAI**				
Model 1	0.000 ± 0.001	-0.002	-0.001,0.001	0.92
Model 2	0.000 ± 0.001	-0.003	-0.001,0.001	0.90
Model 3	-0.000 ± 0.001	-0.003	-0.002,0.001	0.83
**TyG**				
Model 1	-0.000 ± 0.000	-0.001	-0.001,0.000	0.63
Model 2	-0.000 ± 0.000	0.004	-0.001,0.000	0.72
Model 3	-0.000 ± 0.000	0.023	-0.001,0.000	0.55
**HDL (mg/dl)**				
Model 1	-0.003 ± 0.004	-0.001	-0.010,0.005	0.49
Model 2	-0.002 ± 0.004	-0.005	-0.010,0.005	0.53
Model 3	-0.001 ± 0.004	0.002	-0.010,0.008	0.83
**Cholesterol (mg/dl)**				
Model 1	-0.019 ± 0.017	0.001	-0.052, 0.013	0.25
Model 2	-0.019 ± 0.017	-0.006	-0.052, 0.014	0.26
Model 3	-0.024 ± 0.019	-0.004	-0.061, 0.014	0.21
**Triglycerides (mg/dl)**				
Model 1	-0.009 ± 0.028	-0.002	-0.064,0.047	0.76
Model 2	-0.007 ± 0.028	-0.002	-0.062,0.049	0.82
Model 3	-0.007 ± 0.032	0.005	-0.074,0.053	0.75
**FBS (mg/dl)**				
Model 1	-0.011 ± 0.010	0.000	-0.032,0.009	0.27
Model 2	-0.010 ± 0.010	0.008	-0.030,0.010	0.34
Model 3	-0.019 ± 0.012	0.028	-0.041,0.004	0.11
**WC (cm)**				
Model 1	0.008 ± 0.005	0.004	-0.001,0.018	0.07
Model 2	0.009 ± 0.004	0.241	0.001,0.017	**0.03**
Model 3	0.000 ± 0.005	0.258	-0.009,0.010	0.92
**HC (cm)**				
Model 1	0.012 ± 0.004	0.013	0.004,0.020	**0.01**
Model 2	0.012 ± 0.004	0.089	0.004,0.020	**0.01**
Model 3	0.007 ± 0.005	0.099	-0.003,0.016	0.16
**WHR**				
Model 1	-0.000 ± 0.000	-0.001	-0.000,0.000	0.64
Model 2	-0.000 ± 0.000	0.158	-0.000,0.000	0.73
Model 3	-0.000 ± 0.000	0.163	-0.000,0.000	0.18
**BMI (kg/m**^**2**^**)**				
Model 1	0.003 ± 0.002	0.002	-0.001,0.006	0.14
Model 2	0.003 ± 0.002	0.157	-0.002,0.005	0.10
Model 3	0.002 ± 0.002	0.159	-0.002,0.005	0.40

Values are β coefficients (95% CIs), *n* = 546. Results are from multiple linear regression analyses

Model 1: crude

Model 2: adjusted for age, education, marriage, occupation and smoking

Model 3: additionally, adjusted for energy intake and activity score

^*^P-value is considered significant at < 0.05

Abbreviations: *SD* Standard Deviation, *BMI* Body Mass Index, *WHR* Waist to Hip ratio, *WC* Waist circumference, *HC* Hip Circumference, *SBP* Systolic Blood Pressure, *DBP* Diastolic Blood Pressure, *BAI* Body Adiposity Index, *BRI* Body Roundness Index, *ABSI* A Body Shape Index, *HDL* High-Density Lipoprotein, *TC* Total Cholesterol, *TG* Triglyceride, *VAI* Visceral Adiposity Index, *FBG* Fast Blood Sugar, *TyG* Triglyceride Glucose Index, *LAP* Lipid Accumulation Product

## Discussion

To the best of our knowledge, this is the first study that assessed the association of the Mg intake from diet and body composition in a Middle East country like Iran. We compared the quartiles of the energy-adjusted Mg intake in association with BMI, BRI, BAI, ABSI, VAI, LAP, and TyG index in a sample of Iranian adults. Our findings revealed that the dietary intake of Mg was not significantly associated with any of the obesity indicators mentioned above neither in men nor in women, after adjusting for potential confounders, so it did not support the idea that alternation in Mg intake might affect weight and weight-related indices.

In contrast to our findings, a 30- years longitudinal study among 5115 American young adults aged 18–30 years, indicated that Mg intake is inversely associated with BMI [20]. Another population-based probabilistic survey on 1573 Mexican adults showed that increased dietary Mg intake is associated with lower BMI and WC [21]. In a cross-sectional analysis performed on National Health and Nutrition Examination Survey (NHANES) 2007–2014 data, mg intake was negatively correlated with BMI and WC, after adjusting for age and gender [19]. Another cross-sectional study conducted on NHANES 1999–2004 data showed the risk of obesity and central obesity was 20% lower with a 100 mg increase in daily Mg intake [46]. During a 15 years follow-up cohort study among 2247 white men and women, participants in the highest quartile of Mg intake had significantly lower WC, but no meaningful association was found in black men and women and between Mg intake and BMI [47]. The discrepancies between our findings and other studies could be explained by the following reasons. In the present study, Mg supplements were not considered, which can lead to an Mg under-reporting since the people who take nutritional supplements probably have a better diet consisting of seeds and nuts, green vegetables, and high-value legumes [48, 49]. The presence of other confounders also might be a reason for non-significant results. Additionally, Iranian adults may have a higher variance in consuming Mg-rich foods. Besides that, our study had a relatively smaller sample size than previous ones. No research was conducted about the association of Mg intake and previously mentioned novel anthropometric indices.

Obesity, along with type 2 diabetes and metabolic syndrome share a series of pathological pathways that lead to inflammation of the human body [50]. As a result of an unhealthy diet, most obese people have Mg deficiency [51]. Mg depletion may cause chronic inflammation both directly [52] and indirectly by altering intestinal microbiota [53]. In obese people, most of the daily energy intake comes from sugary foods and refined grains [54]. The bioavailability of the Mg mainly depends on the form of the consumed food. The element has a much higher concentration at unrefined whole grains and unprocessed vegetables and fruits compared to processed foods. Additionally, the content of Mg in the edible parts of the cereals and some vegetables is lower than that in non-edible parts [55]. In general, Mg is present in fruits, vegetables, whole grains, legumes, nuts, milk, fish, meat, breakfast cereals, and tap, mineral, and bottled waters [56].

The strength of this study is the relatively large nationally representative sample size and utilizing new indices to explain obesity and body composition. In addition, all information was collected with trained nutritionists using validated questionnaires, reducing any probable error. Also, we acknowledge this is the first study that analyzed the association between Mg consumption and novel body composition indices. Furthermore, we separately analyzed men and women, as there is a clear difference in body composition between the sexes. Furthermore, energy intake was controlled in our study. However, our study has some noticeable limitations. Firstly, this study is cross-sectional in design, and the risk of unmeasured confounders from a large number of dietary, lifestyle, and environmental factors is high, so we cannot estimate the causal relationships of the associations between dietary intake and obesity, thus slightly straying the results to non-significant. Secondly, Mg intake that we extracted was solely based on the consumed meals and not from supplements, which may add up to a big part of daily Mg intake in some individuals, since because of agronomical factors and food processing, the Mg content of foods like fruits and vegetables has decreased significantly [57]. This may end in a high variance in intake which may affect the results. Furthermore, while the FFQ is a typical tool for assessing long-term dietary intake, estimates of food consumption from an FFQ are not exactly accurate, and measurement error is always a possibility [58]. Lastly, since most of the participants were housekeeping women, mean dietary intake might not accurately represent all members of the Iranian society. Also because of lower activity and differences in sleep pattern, the body composition can be greatly affected, expanding the confounders and intensifying the lack of association found in our study. This highlights the necessity for more investigation to authenticate the results across either sex with more diverse occupations.

## Conclusion

In conclusion, no significant association was found between dietary Mg intake and indicators of body composition in healthy Iranian adults. The authors suggest that further prospective cohort studies are warranted to clarify the results.

## Data Availability

The datasets analyzed during the current study available from the corresponding author on reasonable request.

## Abbreviations

BMI: Body Mass Index
WHR: Waist to Hip ratio
WC: Waist circumference
HC: Hip Circumference
SBP: Systolic Blood Pressure
DBP: Diastolic Blood Pressure
BAI: Body Adiposity Index
BRI: Body Roundness Index
ABSI: A Body Shape Index
HDL: High-Density Lipoprotein
TC: Total Cholesterol
TG: Triglyceride
VAI: Visceral Adiposity Index
FPG: Fast Plasma Glucose
TyG: Triglyceride-Glucose Index
LAP: Lipid Accumulation Product
